# New Horizons in Short Children Born Small for Gestational Age

**DOI:** 10.3389/fped.2021.655931

**Published:** 2021-05-13

**Authors:** Irène Netchine, Manouk van der Steen, Abel López-Bermejo, Ekaterina Koledova, Mohamad Maghnie

**Affiliations:** ^1^Sorbonne Université, INSERM, UMR_S938 Centre de Recherche Saint Antoine, APHP, Hôpital Armand Trousseau, Explorations Fonctionnelles Endocriniennes, Paris, France; ^2^Department of Paediatrics, Subdivision of Endocrinology, Erasmus University Medical Centre, Rotterdam, Netherlands; ^3^Girona Biomedical Research Institute, Dr. Josep Trueta Hospital, Girona, Spain; ^4^Global Medical Affairs Department, Merck KGaA, Darmstadt, Germany; ^5^Department of Pediatrics, Institute for Research, Hospitalization and Health Care (IRCCS) Children's Hospital Giannina Gaslini, Genova, Italy; ^6^Department of Neuroscience, Rehabilitation, Ophthalmology, Genetics, Maternal, and Child Health, University of Genova, Genova, Italy

**Keywords:** short stature, small for gestational age, Silver-Russell syndrome, GH treatment, puberty, metabolic abnormalities

## Abstract

Children born small for gestational age (SGA) comprise a heterogeneous group due to the varied nature of the cause. Approximately 85–90% have catch-up growth within the first 4 postnatal years, while the remainder remain short. In later life, children born SGA have an increased risk to develop metabolic abnormalities, including visceral adiposity, insulin resistance, and cardiovascular problems, and may have impaired pubertal onset and growth. The third “360° European Meeting on Growth and Endocrine Disorders” in Rome, Italy, in February 2018, funded by Merck KGaA, Germany, included a session that examined aspects of short children born SGA, with three presentations followed by a discussion period, on which this report is based. Children born SGA who remain short are eligible for GH treatment, which is an approved indication. GH treatment increases linear growth and can also improve some metabolic abnormalities. After stopping GH at near-adult height, metabolic parameters normalize, but pharmacological effects on lean body mass and fat mass are lost; continued monitoring of body composition and metabolic changes may be necessary. Guidelines have been published on diagnosis and management of children with Silver-Russell syndrome, who comprise a specific group of those born SGA; these children rarely have catch-up growth and GH treatment initiation as early as possible is recommended. Early and moderate pubertal growth spurt can occur in children born SGA, including those with Silver-Russell syndrome, and reduce adult height. Treatments that delay puberty, specifically metformin and gonadotropin releasing hormone analogs in combination with GH, have been proposed, but are used off-label, currently lack replication of data, and require further studies of efficacy and safety.

## Introduction

Children born small for gestational age (SGA) are defined as having a birth weight and/or length standard deviation score (SDS) of < −2, based on data from an appropriate reference population (1). There are multiple underlying causes of the reduced growth and, as a result, the classification constitutes a heterogeneous group of patients (2). Causes can include nutritional, hormonal, vascular, genetic and epigenetic factors, and the classification includes those who may have experienced intrauterine growth retardation and who have syndromic conditions such as Silver-Russell syndrome (SRS) (2, 3). Approximately 85–90% of children born SGA experience spontaneous catch-up growth within the first 2 years and then maintain height comparable with their peers (1, 4–6), although a small number of very pre-mature (gestational age <29 weeks) infants born SGA may show catch-up only from 2 to 4 years (7, 8). Lack of catch-up growth and persistent short stature, and also excessive rapid weight gain in early life, is associated with subsequent impaired metabolism, including visceral adiposity, hypercholesterolemia, type 2 diabetes and cardiovascular disease (8–15), as well as psychosocial problems (15–17). Children born SGA also tend to have early onset and faster progression of puberty, with accelerated bone maturation, and early adrenarche (18–20).

Abnormalities of the GH–IGF-I axis occur in children born SGA with persistent short stature, with up to 60% reported to have reduced 24-h GH profile and/or low stimulated GH peak (2, 21–25). While GH levels range from below normal to above normal, there often appears to be GH resistance, with IGF-I concentrations also varying from below to above normal (24–26). However, short children born SGA who have low IGF-I concentrations should be tested for GH deficiency, while those with high IGF-I should be tested for defects of the IGF receptor gene, *IGF1R* (27, 28). GH administration is an approved treatment for children born SGA and who remain short, with initiation from 4 years of age in Europe, due to the continuing potential for spontaneous catch-up growth, although from 2 years of age for formulations licensed in the USA (2, 8, 29). GH therapy increases height velocity, height SDS and adult height in short children born SGA, and also influences other metabolic factors in these children (2, 9, 30–34). The pubertal timing and the quality of the growth spurt affect adult height achieved and, therefore, influencing pubertal maturation may help to optimize height outcome (3, 30, 35).

The present report is based on one of the sessions from a meeting on Growth and Endocrine Disorders in Pediatrics, held in Rome, Italy, sponsored by Merck KGaA, Germany. The meeting session was planned to examine the treatment of children born SGA, particularly in terms of metabolic and pubertal effects.

## Effect of Being Born SGA on Growth and Metabolism in Adolescence and Young Adulthood

It has been established for many years that children born SGA, particularly with low birth weight, are prone to develop type 2 diabetes mellitus and cardiovascular disease (36, 37). The exact mechanism of the association of SGA with diabetes and cardiovascular disease is unknown, although various hypotheses have been proposed, such as a mis-match between the prenatal and postnatal environments. Fetal nutrition may be poor whereas neonatal nutrition may be better, resulting in low birth weight and subsequent rapid weight gain, which may affect pancreatic function (38), possibly through epigenetic changes in DNA methylation (39). Children born SGA are at risk for rapid development of decreased insulin sensitivity compared with those born appropriate for gestational age (AGA), particularly in those who have catch-up growth (40–43). Initially, mean fasting insulin concentration is lower in children born SGA than AGA, but by 3–4 years the mean is greater in the children born SGA, with increasing insulin resistance. Children born SGA also develop altered body composition, and by 4 years of age they may have significantly greater abdominal fat mass and percentage body fat, and lower lean body mass (2, 10, 43, 44). They also tend to have less subcutaneous fat, but the same amount of visceral fat as children born AGA, with an increased ratio of visceral to subcutaneous fat (2, 44).

In a study of young adults born SGA, fat mass in those who remained short was not significantly different from that of peers born AGA, while those born SGA with catch-up growth had slightly, though not significantly, higher fat mass (45). No differences in trunk fat or limb fat mass were found. However, lean body mass was lower in the young adults born SGA, whether remaining short or with catch-up growth, compared with those born AGA, suggesting that fetal reprogramming had long-lasting consequences that remained into adulthood. Insulin sensitivity, from glucose tolerance tests, was similar in the young adults born SGA who remained short, compared with those born AGA, but was significantly lower in the young adults born SGA with catch-up growth. Thus, body composition and insulin sensitivity may indicate that in children born SGA, metabolic abnormalities at young adulthood are more obvious in those who have catch-up growth rather than those who remain short (40, 43).

Multiple clinical trials in short children born SGA have shown that GH treatment can increase adult height, although long-term surveillance data into adulthood continue to be required (2, 8, 33, 46). Short children born SGA who were treated with GH until near-adult height were followed longitudinally for 5 years after stopping GH treatment (45). During this period without exogenous GH, fat mass increased continually, and lean body mass decreased within the first 6 months and then remained constant; reassuringly, the GH-induced decrease in insulin sensitivity was fully reversed within 6 months after stopping GH treatment, despite the increase in fat mass. Thus, the loss of pharmacological effects of GH when adolescents born SGA stopped GH treatment resulted in adverse effects on body composition, but insulin sensitivity improved. Somatic growth is not complete at near-adult height and it is possible, therefore, that further GH treatment could potentially have benefits in the transition period into adulthood, similar to the transition period of adolescents with GH deficiency (47–49).

At 5 years after stopping GH, fat mass was similar in the previously GH-treated young adults born SGA without catch-up growth to that of young adults born AGA, young adults born SGA with catch-up growth, and young adults who remained short and were not treated with GH. Lean body mass was significantly lower in the GH-treated adults than each of the other three groups; these patients were the ones with the shortest length at birth, again suggesting that fetal reprogramming at early life resulted in the lower lean body mass. Insulin sensitivity at 5 years after stopping GH was significantly lower in the adults born SGA and with catch-up growth, compared with those born AGA; the young adults born SGA without catch-up, irrespective of GH treatment, had similar insulin sensitivity to the young adults born AGA (45). This again suggests that metabolic alterations are particularly prevalent in children born SGA who have spontaneous catch-up with rapid weight gain after birth.

Patients born SGA and with persistent short stature generally go to a pediatrician for evaluation for GH treatment, where they should be carefully examined for dysmorphic features, skin abnormalities and head circumference because there may be other factors involved, including possible genetic and organic causes. Children who receive GH treatment should be continually assessed to ensure the original diagnosis is correct, because some genetic causes may only become apparent later in life. The cause of the short stature in children born SGA is a major factor in determining adult outcome of GH treatment (50). Effectiveness of GH treatment should be monitored from height growth, but it is also important to evaluate other factors to ensure safety of treatment. It is recommended to determine IGF-I level at 3–6 months after starting GH treatment and adjust GH dose accordingly unless IGF-I insensitivity is suspected (51), with IGF-I determined annually thereafter. Because GH effects on metabolic parameters of children born SGA are limited and with no long-term adverse effects after stopping treatment, it is debatable whether metabolic and cardiovascular parameters should be evaluated on a regular basis during GH treatment. However, individuals born SGA are at increased risk for metabolic and cardiovascular conditions, and annual assessments of body weight gain, body mass index, blood pressure, and fasting glucose and lipid concentrations seem reasonable, with additional testing when indicated. When GH treatment is stopped at near-adult height, it is most important that patients avoid excess weight gain, but further follow-up depends on potential risk factors, both from the individual medical history and from the family history.

## Diagnosis and Treatment of Silver-Russell Syndrome

Among children born SGA and with persistent short stature, multiple potential causes for the condition have been identified. One specific cause is SRS (OMIM 180860), which is associated with both prenatal and postnatal retardation of growth. SRS was first described in the 1950s; however, guidelines for diagnosis and management were only fully characterized at a consensus conference in 2015, and the guidelines were endorsed by the major endocrine societies around the world (52). It was accepted that, at present, SRS remains based on a clinical diagnosis, and a scoring system for this was developed, known as the Netchine-Harbison system (NH-CSS). It comprises six items, which are being born SGA, postnatal growth retardation, relative macrocephaly at birth, a protruding forehead, feeding difficulties and/or low body mass index in early life, and body asymmetry (53). The scoring system was shown to be highly sensitive (98%) when compared with known molecular identification and it was concluded that a diagnosis of SRS may be made if a child presents with at least four of the six items.

The molecular mechanisms of SRS have been investigated in recent years, and an underlying molecular cause is identified in ~60% of cases (52, 54). The molecular causes involve genetic regions that contain imprinted genes, which are genes that are only expressed on one allele, either maternal or paternal. The most frequently occurring causes of SRS are loss of methylation on chromosome 11p15 (~50% of patients) and maternal uniparental disomy for chromosome 7 (mUPD7; 5–10% of patients). No molecular anomaly was identified in about 40% of patients with a clinical diagnosis of SRS (52–54); however, anomalies in a number of other genes have been identified in recent analyses (55, 56).

The 11p15 region contains two domains that are imprinted: the first is controlled by the *H19*/*IGF2* intergenic differentially methylated region (IG-DMR, previously known as imprinting control region 1, ICR1), which is methylated on the paternal allele and is responsible for expression of *IGF2* during fetal life; the second is controlled by the KCNQ1OT1 TSS-DMR (previously ICR2), which controls expression of the cyclin dependent kinase inhibitor 1C gene (*CDKN1C*) and the maternal allele is methylated. Hypomethylation of the paternal allele at IG-DMR is the most frequently occurring defect in SRS, resulting in diminished fetal IGF-II concentration and an increase in maternal expression of the *H19* long non-coding RNA, leading to fetal growth retardation (52, 54, 57). Duplication of the maternal allele results in overexpression of *CDKN1C*, which causes a break in the cell cycle and acts as a growth repressor, again causing fetal growth retardation (52, 58, 59); *CDKN1C* gain-of-function mutations have also been found to result in SRS (56, 60). Mutations in the oncogenes *HMGA2* and *PLAG1* have also been found in familial cases of SRS, due to disruption of *IGF2* expression (56, 61, 62).

**Figure 1:**
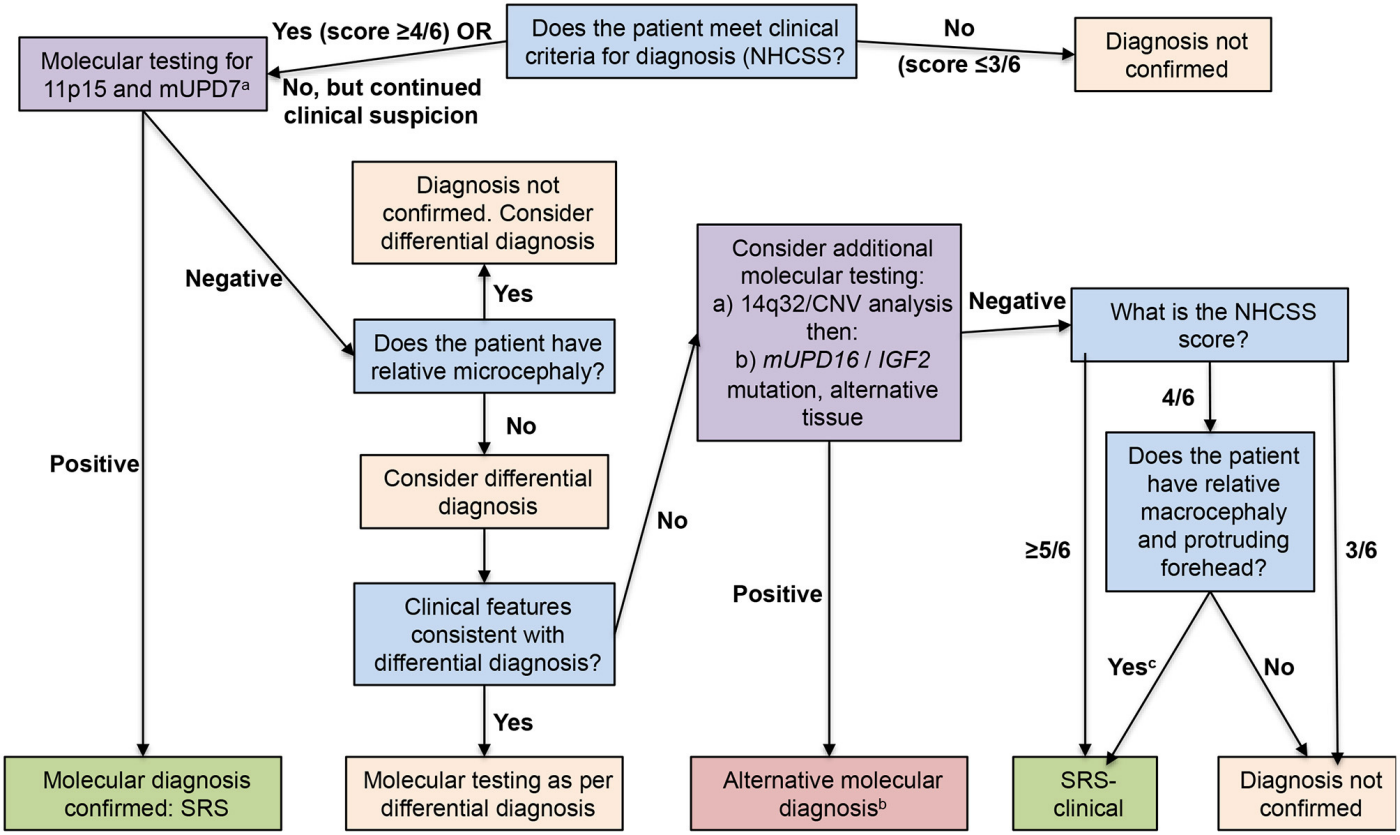
Flow chart for investigation and diagnosis of patients with suspected Silver-Russell syndrome. Blue boxes show diagnostic questions, mauve boxes recommended molecular testing, pink boxes diagnosis not confirmed, and green boxes diagnosis confirmed. Reproduced from: Wakeling et al. (52). ^a^Arrange copy number variant analysis before other investigations if patient has notable unexplained global developmental delay and/or intellectual disability and/or relative microcephaly; ^b^Insufficient evidence at present to determine relationship to Silver-Russell syndrome, with the exception of tissue mosaicism for 11p15 loss of methylation; ^c^Previously known as idiopathic Silver-Russell syndrome.

The consensus conference provided guidelines (52) for investigation of patients with suspected SRS (Figure 1). The first assessment should be whether the scoring system indicates a positive result. If the clinical score is at least 4, then anomalies in methylation of 11p15 or mUDP7 alleles should be examined and the presence of anomalies confirms a diagnosis of SRS. If no anomalies in these alleles are seen, the possibility of a differential diagnosis should be discussed with a clinical geneticist; an indicator of a differential diagnosis is the presence of relative microcephaly, with head circumference SDS less than height SDS and/or weight SDS, which would indicate a number of different syndromes to be considered (52). If the geneticist does not believe there is an evident differential diagnosis, more complex molecular analysis should be carried out, including examination of epimutational disomy at chromosome 14q32, other disomies at mUPD16 and mUPD20 or mutations of *CDKN1C, IGF2, HMGA2*, and *PLAG1* (52, 55, 56, 60–63); a positive result for any of these would provide an alternative close diagnosis. If this further molecular analysis is negative, then a diagnosis of SRS can be retained only if the patient has a score of at least 4, including relative macrocephaly and protruding forehead; the diagnosis is then clinical SRS with no molecular anomaly at present identified.

Once a diagnosis of SRS is confirmed, the nutrition of the patients should be carefully controlled to avoid both under and over nourishment and limit the amount of adipose tissue. In the first years of life, nutritional repletion is recommended, with awareness of possible hazards of rapid postnatal catch-up leading to later metabolic risk. The nutritional control can be determined in children aged <4 years from the Water low classification of weight for height/length (64), with the aim of 75–85% of the expected value, or from a body mass index for children aged 2–4 years, with a target of −2 to −1 SDS. In children older than 4 years, the target depends on the muscle mass, because a reduced muscle mass makes body mass index targets excessive; however, it is important to also try to improve the muscle mass. Long-term studies of adult patients with SRS are limited at present; however, a study in 7 adults with SRS showed fat mass index and trunk fat mass were significantly increased compared with healthy controls (65).

Cognitive function of patient with SRS may be compromised, but with heterogeneity in the effects (66). In children aged 6–16 years, those with SRS due to 11p15 loss of methylation had cognitive scores similar to control children, whereas those with SRS due to mUPD7 had significantly lower cognitive scores. Intellectual functioning in 10 adult patients with SRS due to 11p15 loss of methylation was assessed by Full Scale Intelligence Quotient and was in the average range, but learning disabilities and low self-esteem were perceived by 60% of these adults (67).

Children with SRS are at high risk of hypoglycemia and home monitoring of urinary ketone levels is considered useful to determine which children need intervention (52). A plan should be developed for rapid clinic admission and treatment with intravenous dextrose when the child is ill. Surgery should be carefully planned due to the increased risks of fasting hypoglycemia, hypothermia, and possible difficulties of intubation, and it should be borne in mind that malnourished children with SRS may not heal well following surgery (52, 68, 69).

Most children with SRS do not have catch-up growth and remain short (2, 52), so can benefit from treatment with GH under the SGA indication. GH treatment can not only optimize linear growth, but can also improve body composition, particularly lean body mass, aid psychomotor development and appetite, and reduce the risk of hypoglycemia. However, some patients with SRS have sleep apnea and this should be taken into consideration before and during GH therapy (70). Treatment with GH should start as early as possible. Children with SGA may show catch-up growth and the European approved treatment start is at age >4 years, in contrast to the USA where treatment may start at age >2 years (2, 8). Children with SRS rarely have catch-up growth and the consensus guidelines suggested that GH initiation at 2–4 years of age is preferable (52); similar to any other medication however, the benefit-risk assessment of GH treatment for children with SRS should be evaluated constantly. GH deficiency is not commonly found in children with SRS, but presence or absence does not appear to affect response to exogenous GH treatment (71). Prior to GH treatment, IGF-I levels in children with SRS are normal or elevated, are higher than for patients with other forms of SGA and are higher in patients with 11p15 anomalies than mUPD7 defects, suggesting that there may be an element of IGF-I resistance (72–74). During GH treatment, IGF-I levels increase and may be higher than the normal range, particularly in children with 11p15 IG-DMR hypomethylation; therefore, IGF-I results may be difficult to interpret, although should still be monitored at least yearly (52, 72–74).

Children with SRS can have pre-mature adrenarche, with early accelerated puberty and a rapid acceleration of bone age, irrespective of GH treatment (52, 75, 76). Children should, therefore, be monitored for pre-mature adrenarche and accelerated bone age should be anticipated, especially from mid-childhood onwards. In male children with SRS, however, it may be more difficult to determine puberty start due to reduced testicular size as a symptom of SRS (77, 78). Pre-mature adrenarche, with rapid bone maturation, may reduce the time during which GH can be effective for children with SRS. Therefore, patients may benefit from personalized treatment with a gonadotropin releasing hormone (GnRH) analog to delay puberty and enable continued effects of exogenous GH treatment (52, 74, 77). However, combined GnRH analogs with GH is not currently an approved indication; the consensus report suggested that such treatment should be on an individual basis and further studies are required to examine effects in patients with SRS (52).

## Pubertal Maturation in Patients Born SGA

There remains, at present, a lack of data to determine the concerns of pubertal development in children born SGA. Studies have established that pubertal onset occurs earlier in children born SGA than in children born AGA, although timing appears appropriate for chronological age and height (18, 19, 79, 80). Bone maturation during puberty is accelerated, peak height velocity occurs earlier and for a shorter period of time, fusion of the growth plates occurs earlier and menarche is earlier, which all result in a reduced adult height (19, 79, 80). It is believed that this accelerated pubertal development is related to the rapid weight gain in early childhood, which causes increased visceral adiposity, decreased insulin sensitivity and elevated IGF-I concentrations (81); thus, children born SGA who have catch-up growth have a higher risk of early and accelerated puberty (19). Pubertal alterations have been documented to occur in both sexes, but to be more pronounced in girls than boys (18, 79). GH treatment in short children born SGA has no apparent effect on age of pubertal onset or progression of puberty, irrespective of GH dose (82). There was also no effect of GH on age at menarche or the interval between breast development and menarche in girls.

Management of metabolic and pubertal abnormalities requires careful observation and weight control. Intervention options that have been suggested include treatment with metformin or with a GnRH analog combined with GH, which are currently not approved treatments. In children born SGA who have catch-up growth and a normal height prognosis, a rapid gain in body weight and accompanying visceral adiposity are associated with early and rapidly progressing puberty (43, 83). Therefore, it is important to limit the weight gain in all children born SGA to try to reduce the adverse metabolic and pubertal effects. Metformin is associated with reduced weight gain and has been examined in several off-label studies in children born SGA, particularly in children with low birth weight (84–87). Treatment with metformin caused decreases in central adiposity, insulin resistance and IGF-I levels, which are pathologically associated with early puberty. In children with low birth weight and precocious pubarche, metformin treatment for at least 3–4 years resulted in a delay of menarche by ~1 year, prolonged pubertal growth, and augmentation of adult height by ~4 cm (43, 84, 88).

Similar to the specific group of patients with SRS, off-label use of GnRH analogs to delay puberty during GH treatment has been suggested more generally in various studies in patients born SGA (52, 89), and has been suggested to have no unfavorable effects on metabolic or psychological health at adult height (17, 35). In a study of short children born SGA who were treated with GH but remained short at puberty onset, with a predicted adult height <−2.5 SDS, additional treatment with a GnRH analog was given for 2 years (90). Adult height in these patients was similar to those who were taller at puberty onset and treated with GH alone, suggesting that children born SGA who remained short at puberty onset could benefit from combined GH plus GnRH analog treatment. The study also suggested that, in combination with the GnRH analog, a higher than approved daily GH dose of 2 mg/m^2^ could improve adult height of short children born SGA to a greater extent than the standard dose of 1 mg/m^2^/day. However, IGF-I SDS increased above 2 for 33% of the higher GH dose group vs. 6% of the lower dose group, although IGF-I concentrations were significantly lower in the patients treated with combined GnRH analog plus GH than in those treated with GH alone (90, 91). Percentage trunk fat increased in both groups treated with GnRH analog, fat mass increased in patients receiving GnRH analog plus the lower GH dose and lean body mass increased only in the group treated with the high GH dose (91). However, blood pressure, lipid profile and insulin sensitivity were similar for children born SGA treated with GnRH analog plus GH and those treated with GH alone, indicating no negative effects of the combined treatment on metabolic and cardiovascular parameters. In the children treated with GnRH analog, puberty started within the normal age range, although pubertal duration after stopping GnRH analog was shorter than duration in children treated with GH only (89).

## Conclusions

The majority of children born SGA have catch-up growth within the first few postnatal years and there is currently no evidence to recommend routine investigation of all children born SGA. However, rapid catch-up growth can increase the risk of metabolic syndrome and early puberty; therefore, it is advised that these patients should be followed up by the pediatrician in routine clinical visits to monitor body composition and metabolic parameters. For those children who remain short, GH treatment improves adult height outcome. Regular follow-up during GH treatment is required to ensure effectiveness of the treatment and to re-evaluate the original diagnosis to ensure it is correct. After stopping GH treatment at near-adult height, metabolic parameters return to normal and long-term GH treatment appears to have no unfavorable effects on metabolic and cardiovascular health in adulthood. However, the pharmacological effects on body composition are lost when GH treatment is stopped, and fat mass increases while lean body mass decreases. Continued follow-up after stopping GH treatment depends on potential risk factors, although patients should always be cautioned against excess weight gain.

Children born SGA who show specific defined characteristics, according to recent guidelines, may be assessed for a diagnosis of SRS, particularly on the basis of molecular evaluations. Children diagnosed with SRS almost always remain short and benefit from GH treatment starting as early as possible. However, there is currently limited information regarding adult patients with SRS, although body composition may be impaired, with increased fat mass index and trunk fat, and it is important to maintain contact with such patients. Case studies of adults with SRS due to 11p15 hypomethylation have reported problems of hypertension, dilated cardiomyopathy, type 2 diabetes mellitus and hypercholesterolemia, but there is little information to indicate generalization of such conditions. Further studies are required to establish a cohort of adults with SRS to examine the consequences with regard to the long-term metabolic risks.

Children who are born SGA and remain short, including those with SRS, tend to have earlier and rapidly progressing puberty, with faster bone maturation and a shorter period of pubertal peak height velocity, associated with metabolic abnormalities such as visceral adiposity. As a consequence, these children remain short at adult height. However, evidence for treatment is limited, with few longitudinal clinical trials. There is a lack of replication studies of off-label use of metformin to reduce adiposity and delay puberty in short children born SGA, and metformin is associated with gastrointestinal adverse events. There are also limited studies of off-label use to delay puberty with a GnRH analog, for at least 2 years, in combination with GH, in children born SGA who remain short at onset of puberty. The studies that combined a GnRH analog with GH treatment indicated a possible increase in adiposity with GnRH analog treatment, although this was reduced after GnRH analog was stopped. In children born SGA with a low predicted adult height, combined GnRH analog and GH treatment may improve adult height outcome, without long-term negative metabolic or cardiovascular effects. Thus, evidence for current therapies to modulate pubertal growth in children born SGA is limited and further studies are required.

## Author Contributions

All authors were involved in the preparation and critical revision of the current work for important intellectual content, gave final approval of the version of the manuscript to be published, and have agreed to be accountable for all aspects of the work in ensuring that questions related to the accuracy or integrity of any part of it are appropriately investigated and resolved.

## Conflict of Interest

EK is an employee of Merck KGaA, Darmstadt, Germany. The remaining authors declare that the research was conducted in the absence of any commercial or financial relationships that could be construed as a potential conflict of interest.
